# An Evaluation of Three Kinematic Methods for Gait Event Detection Compared to the Kinetic-Based ‘Gold Standard’

**DOI:** 10.3390/s20185272

**Published:** 2020-09-15

**Authors:** Nicole Zahradka, Khushboo Verma, Ahad Behboodi, Barry Bodt, Henry Wright, Samuel C. K. Lee

**Affiliations:** 1 Biomechanics and Movement Science Program, University of Delaware, Newark, DE 19713, USA; nzahrad1@jhu.edu (N.Z.); vkhush@udel.edu (K.V.); ahadbeh@udel.edu (A.B.); 2 Department of Physical Therapy, University of Delaware, Newark, DE 19713, USA; henryw@udel.edu; 3 Biostatistics Core Facility, College of Health Sciences, University of Delaware, Newark, DE 19713, USA; babodt@udel.edu; 4 Shriners Hospitals for Children, Philadelphia, PA 19140, USA

**Keywords:** gait event detection, wearable sensors, gait analysis

## Abstract

Video- and sensor-based gait analysis systems are rapidly emerging for use in ‘real world’ scenarios outside of typical instrumented motion analysis laboratories. Unlike laboratory systems, such systems do not use kinetic data from force plates, rather, gait events such as initial contact (IC) and terminal contact (TC) are estimated from video and sensor signals. There are, however, detection errors inherent in kinematic gait event detection methods (GEDM) and comparative study between classic laboratory and video/sensor-based systems is warranted. For this study, three kinematic methods: coordinate based treadmill algorithm (CBTA), shank angular velocity (SK), and foot velocity algorithm (FVA) were compared to ‘gold standard’ force plate methods (GS) for determining IC and TC in adults (*n* = 6), typically developing children (*n* = 5) and children with cerebral palsy (*n* = 6). The root mean square error (RMSE) values for CBTA, SK, and FVA were 27.22, 47.33, and 78.41 ms, respectively. On average, GED was detected earlier in CBTA and SK (CBTA: −9.54 ± 0.66 ms, SK: −33.41 ± 0.86 ms) and delayed in FVA (21.00 ± 1.96 ms). The statistical model demonstrated insensitivity to variations in group, side, and individuals. Out of three kinematic GEDMs, SK GEDM can best be used for sensor-based gait event detection.

## 1. Introduction

Gait analysis is commonly performed to characterize healthy walking and quantify deviations that may exist due to pathology or injury [1,2,3,4,5,6,7,8,9]. The gait cycle is defined as the time interval between two successive occurrences of one of the repetitive events of walking [10], typically beginning from initial contact (IC) of the foot to the successive IC. The timing of IC and terminal contact (TC), referred to as heel strike and toe off in healthy populations, is necessary to mark the transition between stance and swing phases of gait. These two events are essential to analyze temporal gait parameters, such as stride time, periods of single and double support [11] and to compare joint angles, forces, and moments across multiple strides [12]. The division of the gait cycle also allows clinicians to evaluate deviations in pathologic gait, and improvements achieved with rehabilitation, by providing a clear description of the typical behavior of the lower extremity during each phase of the gait cycle.

A gait or motion capture laboratory, equipped with force plates or instrumented treadmills and cameras, is the “gold standard” [5,13,14,15] for providing complete biomechanical analysis of the spatiotemporal, kinematic and kinetic parameters of gait, of which, gait event detection (GED) is a necessary component. Gait laboratories, however, require sophisticated motion capture systems, a well-trained team, abundant time and resources for analysis [10,16,17]. Although considered the standard for gait biomechanical analysis, laboratory-based analysis is not fully representative of walking in daily life situations, particularly for patient populations [18,19,20]. Recently, there is a boom in the development of video and wearable sensor-based techniques for gait analysis in ‘real world’ scenarios outside of the typical instrumented motion analysis laboratory [21,22,23,24,25]. Such systems do not use kinetic data from force plates in their algorithms to determine gait events such as IC and TC. Rather, they make estimations of gait events from video data or sensor signals, such as those from gyroscopes [21,24,26,27], accelerometers [28,29,30,31], EMG [32,33], and force sensitive resisters [34,35].

Gait event detection from sensor data has also been leveraged in sensor-based rehabilitation systems. Detection of gait events such as IC and TC are crucial when considering use of orthotic or therapeutic interventions, especially in functional electrical stimulation (FES) [29,34,35,36] and rehabilitation robotic systems [37,38,39], that use gait events to synchronize stimulation delivery or actuator activation to particular gait phases. Although detection delays and timing estimation errors are inherent in kinematic-based gait phase detection methods when compared to the “gold standard” method (gait events from force plate data), the method used to estimate gait events themselves may impact the timing differences observed. Quantifying gait event delays [5] and subsequently providing compensation algorithms for gait event timing errors [40] are crucial for applications in which gait events serve to trigger assistive applications. This approach allows for appropriate correction and compensation of gait event detection errors to minimize timing errors for the assistive applications [40,41].

Kinematic methods use different variables to estimate gait events, therefore, each method may introduce their own systematic characteristic errors in estimating timing of gait events when compared to events determined from force plate data. Thus, analysis of various kinematic methods in detecting gait events is necessary for determining repeatability, accuracy, and reliability to implement in non-laboratory-based gait analysis systems, such that they can be used as standard tools for assessment or to provide gait events as inputs to rehabilitation systems. Therefore, we utilize the gold standard technique (motion capture laboratory and force plates) of gait event detection to evaluate the differences that three different commonly used kinematic methods have on gait event detection (GED) timing. The purpose of this manuscript is to (1) quantify timing accuracy of three kinematic methods; the coordinate-based treadmill algorithm (CBTA) [25], the shank angular velocity algorithm (SK) [24] and the foot velocity algorithm (FVA) [11]; to determine IC and TC gait events compared to the established ‘gold standard’ kinetic-based method (GS), and (2) through statistical modeling, demonstrate that kinematic and therefore sensor-based methods for detecting IC and TC events are insensitive to covariate influences, i.e., subject group differences, person-specific variability, side-to-side gait differences, and the kinematic-based GEDM used to derive the gait events. Finally, we make the case for one particular kinematic-based method that can best be used for sensor-based gait event detection.

## 2. Materials and Methods

### 2.1. Gait Event Detection Methods (GEDM)

**Figure 1:**
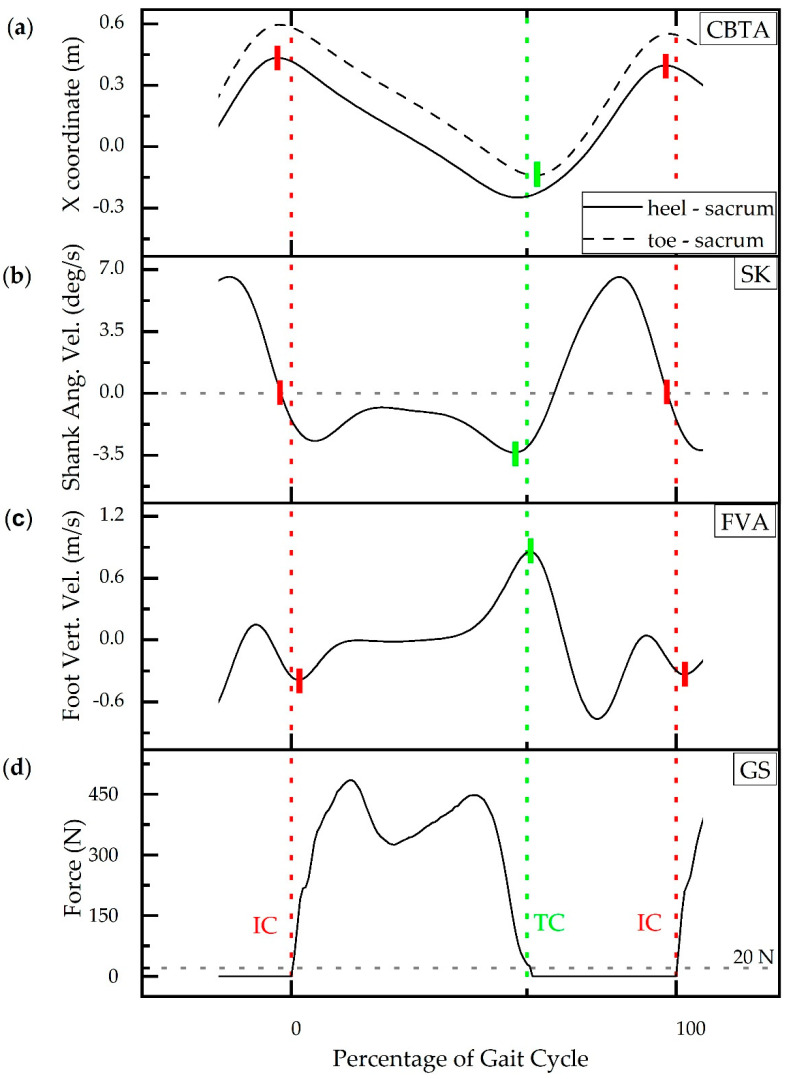
Representative kinematic and kinetic signals for four gait event detection methods (GEDM) during walking for a typically developing child. Kinematic GEDM: (**a**) the resultant X coordinate formed by the subtraction of the X coordinate of the sacral marker from the X coordinate of the heel marker (solid) and resultant X coordinate formed by the subtraction of the X coordinate of the sacral marker from the X coordinate of the toe marker (dashed) (CBTA); (**b**) shank angular velocity (SK); (**c**) vertical foot velocity (FVA); (**d**) ‘gold standard’ force plate method (GS). GS kinetic algorithm-based initial contact (IC) detection (red dotted line) and terminal contact (TC) detection (green dotted line). Solid red and green hash marks indicate IC and TC detection estimates by the respective kinematic methods.

Gait event detection based on force plate data is considered the gold standard (GS) [5,13,14]. Initial Contact (IC) is represented by the initiation of force detection upon contact, and terminal contact (TC) is the return of force to zero as contact with the plate ceases (Figure 1d). A threshold of 20N was applied to the force plate signal in the z-direction to determine the timing of IC and TC [12]. The event times corresponding to the kinetic method (GS) is used to compare the accuracy of IC and TC detection times of three kinematic methods. The three kinematic gait event detection methods (GEDM) used to estimate IC and TC from motion capture data are the coordinate-based treadmill algorithm (CBTA) [25], the shank angular velocity algorithm (SK) [24], and the foot velocity algorithm (FVA) [11] (Figure 1).

The CBTA method plots the sinusoidal curves of the resultant X coordinates formed by the subtraction of the X coordinate of the sacral marker from the X coordinate of the heel marker and toe markers, respectively, as a function of time [25]. IC is defined as the maximum value of the resultant X coordinate formed by subtraction of the X coordinate of the sacral marker from the X coordinate of the heel marker (Figure 1a—solid), while TC is defined as the minimum value of the resultant X coordinate formed by subtraction of the X coordinate of the sacral maker from the X coordinate of the toe marker (Figure 1a—dashed). The SK method utilizes the negative zero crossing of shank angular velocity to detect IC and the second trough value to detect TC (Figure 1b) [24]. The FVA method uses the vertical velocity of the foot center of gravity to detect initial contact and terminal contact with the first troughs and the subsequent peak representing IC and TC, respectively (Figure 1c) [11].

### 2.2. Experimental Protocol

Gait analysis was performed on seventeen participants during walking to evaluate the effect of GEDM on gait event timing. Temple University Institutional Review Board (IRB) approved consent was obtained from six adults (AD) (age 24–36 year), and parental consent and child assent were obtained from five typically-developing (TD) children (age 10–16 year) and six children with spastic diplegic cerebral palsy (CP) (age 12–18 year, Gross Motor Function Classification System Level II (*n* = 3) and III (*n* = 3)) prior to participation (protocol # 20459). Group characteristics (mean ± SD) included—height: AD: 1.70 ± 0.08 m, TD: 1.57 ± 0.12 m, CP: 1.62 ± 0.10 m; weight: AD: 72.35 ± 13.97 kg, TD: 47.35 ± 14.05 kg, 56.07 ± 6.44 kg; and self-selected walking speed: AD: 0.92 ± 0.18 m/s, TD: 0.99 ± 0.17 m/s, CP: 0.72 ± 0.15 m/s.

Kinematic and kinetic data were collected while individuals walked on an instrumented treadmill at self-selected speeds. Participants wore standardized study-provided footwear and a modified Cleveland Clinic marker set was used to capture kinematic data. An eight-camera system (Motion Analysis Corporation, Santa Rosa, CA, USA), with a sampling frequency of 128 Hz, and two force plates (Bertec Corporation, Columbus, OH, USA), with a sampling frequency of 3200 Hz, were used to record 30 s walking data after a treadmill-walking accommodation period [25]. Marker and analog data were processed retrospectively in Visual 3D and filtered using a Butterworth low pass filter with cutoff frequencies of 6 and 25 Hz, respectively. After a lower extremity model was applied to each dataset, detection times associated with IC and TC were determined using four GEDM: GS (kinetic), CBTA, SK and FVA.

### 2.3. Statistical Analysis

#### 2.3.1. Overall Performance

Gait event time differences between the kinematic methods (CTBA, SK, and FVA) and kinetic method (GS) were summarized using mean ± SE and root mean square error (RMSE). All steps were analyzed within a given group. The average step count was AD: 106, TD: 88, and CP: 99 with the median number of steps being AD: 106, TD: 110, and CP: 106. Thus, the step counts were reasonably balanced across groups. Gait detection reliability (GDR) represents the accuracy of a detection method in detecting a gait event (IC/TC) compared to the number of gait cycles evaluated. GDR is the ratio of number of gait events divided by number of gait cycles. GDR was calculated for each detection method (GS, CBTA, SK, and FVA) and reported as a percentage.

#### 2.3.2. Covariates

Multilevel random coefficient models were developed to systematically evaluate the influence of covariates on gait event time differences between the kinematic methods and GS. Variables investigated were group, gait event, side, and subject. Models were developed independently for each of the kinematic GEDMs (CBTA, SK, and FVA), which we will refer to as the principal predictors, to fit the GS. All random coefficients models were developed in JMP^®^Pro 14.3.0 (SAS Institute, Inc., Cary, NC, USA).

Initially, each model allowed adjustments for additive covariates indicating group (CP, TD, AD), gait event (IC, TC), and side (left, right) as well as adjustments to slope and intercept according to each subject. Additive covariates were suggested by exploratory graphs. The full model, expressed in terms of GEDM, is as follows:

$${\mathrm{Standard}}_{ij}=\beta_{1}+\beta_{2}{\mathrm{GEDM}}_{ij}+\beta_{3}{\mathrm{AD}}_{ij}+\beta_{4}{\mathrm{CP}}_{ij}+\beta_{5}{\mathrm{IC}}_{ij}+\beta_{6}{\mathrm{SL}}_{ij}+b_{i1}+b_{i2}{\mathrm{GEDM}}_{ij}+\varepsilon_{ij}$$

Indices *ij* denote subject *i* and observation *j*. AD and CP are dummy variables 0/1 indicating a subject group is AD (1), CP (1); otherwise, AD = CP = 0 and the model reverts to group TD. Similarly, IC (1) indicates initial contact and SL (1) indicates the left side. Marginal model $\beta^{\prime }s$ are estimated for each predictor. The *b’s* are random coefficients associated with each subject. They introduce a conditional (to subject) adjustment of the fit as to intercept and slope coefficients. The error term represents the model error within each subject. The full model is *conditional* on subject. The *marginal* model suppresses adjustment to subject and provides the model averaged over subjects.

#### 2.3.3. Statistical Modeling

This full random coefficient model, which leveraged all additive covariates and subject-specific coefficients, was used as the starting point to determine the best model involving each of the principal predictors [42]. Best is a trade-off between model parsimony and fit. A typical process was followed: (1) fit the full model, (2) evaluate departures from model assumptions, screening some data as required, (3) assess model significance and fit, (4) determine contribution and significance of model coefficients, reducing the model by removing terms as indicated, and (5) iterating on steps (3) and (4) as needed. Fit diagnostics and model development for this dataset are detailed in the results (Section 3.2).

## 3. Results

**Figure A1:**
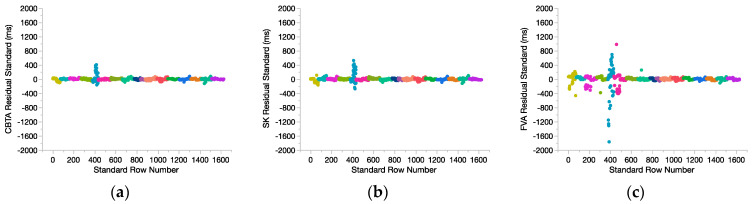
Gait event time residuals (ms) when detected with different kinematic methods for each subject. Blue data points illustrate one individual with the largest residuals in each plot. (**a**) coordinate-based treadmill algorithm (CBTA); (**b**) shank angular velocity algorithm (SK); (**c**) foot velocity algorithm (FVA).

Gait events from 16 out of 17 subjects were included in the analysis and 1502, 1506, and 1480 gait events were analyzed for CBTA, SK, and FVA, respectively. Half of events included in analysis were IC. One subject in the CP group was identified as an outlier and excluded from analysis. This individual had inconsistent scissoring across midline, exhibited dragging of the toes, and had a lower functioning GMFCS level III. These gait deviations contributed to misdetections in the standard kinetic force plate detection method as well as for the three kinematic methods. Refer to the Appendix for further discussion of this subject and how this subject’s data affected statistical analysis (Figure A1).

### 3.1. Overall Performance

The mean ± SE and RMSE were calculated for the detection time difference of IC and TC between kinematic GEDMs and GS. The mean assigns a direction to the detection difference; positive values indicate a delay in event detection and negative values indicate premature event detection of the kinematic GEDM. The RMSE values allow comparison of kinematic GEDM differences to the GS independent of direction.

**Figure 2:**
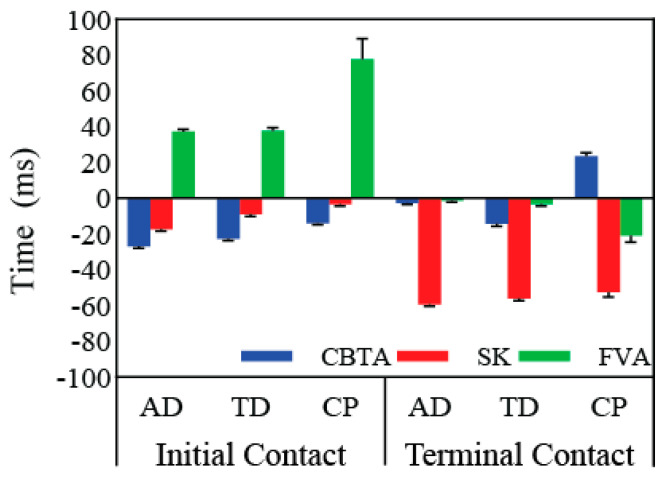
Initial and terminal contact time difference (mean ± SE) of kinematic versus kinetic gait event detection methods (GEDM) for each group. Positive values indicate a delay in event detection while negative values indicate premature detection of the kinematic GEDM. CBTA: coordinate-based treadmill algorithm; SK: shank angular velocity algorithm; FVA: foot vertical acceleration algorithm; AD: adult; TD: typically developing children; CP: children with cerebral palsy.

On average, CBTA and SK detected gait events earlier (CBTA: −9.54 ± 0.66 ms, SK: −33.41 ± 0.86 ms) while FVA had delayed GED (FVA: 21.00 ± 1.96 ms) compared to the GS. Average IC detection difference was −21.54 ± 0.66 ms (CBTA), −10.45 ± 0.74 ms (SK), and 49.50 ± 3.43 ms (FVA). Average TC detection difference was 2.47 ± 0.96 ms (CBTA), −56.20 ± 1.02 ms (SK), and −6.88 ± 1.33 ms (FVA) (Table 1). Average event timing of kinematic GEDMs also varied between groups (Figure 2).

The RMSE values for CBTA, SK, and FVA were 27.22, 47.33, and 78.41 ms, respectively, and suggest that CBTA most closely approximates the gold standard. Support for this claim is further drawn from forward stepwise modeling with all three predictors available, where the algorithm clearly prefers CBTA, to SK, to FVA in the order of entry into the model. Average IC RMSE was similar between CBTA (28.08 ms) and SK (22.81 ms); however, SK TC RMSE was ~36 ms larger than CBTA TC RMSE (26.30 ms). FVA had an average IC RMSE of 105.03 ms and TC RMSE of 36.91 ms. RMSE of kinematic GEDMs also varied between groups (Table 2).

Gait detection reliability (GDR) was evaluated for 639, 437, and 495 gait events in AD, TD, and CP, respectively. Gait detection reliability is listed for each GEDM in Table 3. On average, the GDR of the gold standard (GS) was 99.7% detecting 1566 out of 1570 gait events. The CBTA, SK, and FVA detected 1503, 1509, and 1483 out of 1570 gait events respectively. Average GDR was highest for SK (96.11%) followed by CBTA (95.73%) and FVA (94.46%).

### 3.2. Statistical Modeling

#### 3.2.1. Fit Diagnostics: Data Reduction

Initial models for fitting GS to CBTA, SK, and FVA were based on 1555, 1557, and 1530 records, respectively, taken from 17 subjects (6 AD, 6 CP, 5 TD). Performance of the marginal model, expressed in terms of RMSE for prediction, yielded for CBTA (31.48 ms), SK (43.17 ms), and FVA (116.45 ms), with marginal coefficients for the three principal predictors scarcely discernable from a perfect fit (slope = 1) with CBTA (0.99997), SK (1.00008), and FVA (0.99937), (*p* < 0.0001). However, model diagnostics identified a pattern of residuals inconsistent with model assumptions, largely attributable to a single subject in the CP group (Figure A1). This subject was excluded from analysis (Appendix A).

**Figure 3:**
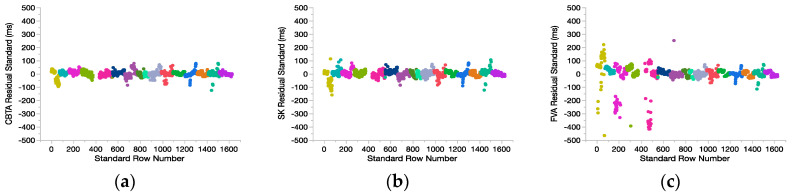
Gait event time residuals (ms) when detected with different kinematic methods for each subject: (**a**) coordinate-based treadmill algorithm (CBTA); (**b**) shank angular velocity algorithm (SK); (**c**) foot velocity algorithm (FVA).

Full models for fitting GS to CBTA, SK, and FVA were based on 1502, 1506, and 1480 records, respectively, taken from 16 subjects (6 AD, 5 CP, 5 TD). RMSE was revised for CBTA (20.00 ms), SK (24.14 ms), and FVA (70.02 ms) with significant marginal coefficients for CBTA (0.99999), SK (0.99994), and FVA (0.99989), (*p* < 0.0001). Patterning in residuals is absent for CBTA and SK, but exists in FVA for certain subjects with CP demonstrating large residuals (Figure 3c). Normal quantile plots for residuals of each model show well-behaved, symmetric distributions but with slightly heavier tails than a normal distribution.

#### 3.2.2. Model Development

Table 4 summarizes the progression of the analysis toward a final model, grouped by principle predictor. Full models for each principal predictor, with one subject excluded, are reported with model coefficient statistics, and marginal and conditional RMSE. Only the coefficients for the principal predictor and intercept are reported in each full model for comparison to subsequent models.

The full model performance, with no covariates eliminated, performed well for each of the three kinematic gait event detection methods. Note coefficients for the principal predictors are nearly a perfect 1 and *marginal* RMSEs on the order of $10^{2}\;\mathrm{ms}$. The smaller *conditional* RMSE reflects the advantage of including subject specific adjustments to the intercept and principal predictor slope. However, including subject specific adjustment, the effective change on RMSEs is on the order of $10^{1}\;\mathrm{ms}$ demonstrating relative insensitivity of subject specific variability on the GEDMs to detect gait events. For example, the full FVA model estimates subject variability (standard deviation) as 27.56 ms and $5\times 10^{-4}$, respectively, for the intercept and slope.

Among the covariates, only IC was statistically significant and with a potentially clinically relevant effect size in models involving each of CBTA, SK, and FVA. For CBTA, the coefficient for the CP indicator was significant (*p* = 0.0164) as well as the indicator for the left side (*p* < 0.0001). The CBTA method in particular, demonstrated errors in determining IC/TC timing for individuals with CP and particularly with gait differences for the left side of these individuals. The effect sizes (adjusting the model up or down), however, were only −22.4 and −6.0 ms, respectively. Because of these small magnitudes and in deference to a parsimonious model, the exclusion of group and side caused little change in the overall RMSE. Therefore, these two terms were not included in subsequent models using CBTA. Only gait event (IC) was initially carried forward as it was common to models based on all three principal predictors.

Reduced models, (CBTA, SK, FVA) + IC retained only the principal predictors, IC, and random coefficients. IC was consistently significant in the full models and remained so in the reduced models. The RMSE necessarily grows with the paring of model terms and is most readily apparent in the small increase in marginal and conditional RMSEs when moving from the full CBTA model to the reduced model (CBTA+IC) (Table 4). Final models, listed by principal predictor, contain only the principal predictor and random coefficients. Relative to the reduced models the RMSE grew <10 ms for each model as can be seen in Table 4 RSMEs when moving from the reduced to final models.

## 4. Discussion

Three kinematic gait event detection methods (GEDM) for detecting gait events (IC and TC) during walking were evaluated in three populations (AD, TD, and CP). Comparisons of gait event detection (GED) time and gait detection reliability (GDR) of each kinematic GEDM were made to the gold standard (GS) of using force plates (kinetic) data to detect events. Our approach helps to characterize the errors in GED among different kinematic methods such that, with compensatory algorithms for timing errors, non-laboratory-based gait analysis systems using such techniques can be equivalent to gold standard laboratory systems. A novelty of our work is the advantage of applying the four GEDMs to the same dataset for the direct comparison of GEDM accuracies rather than comparing across different studies and validation techniques. Thus, the present work assists in the validation of various kinematic methods in detecting gait events, and to our knowledge, is the first to use a statistical model demonstrated insensitivity of the GEDMs to variations in group, side, and individuals.

Gait event detection timing of CBTA, SK, and FVA were compared to previously reported accuracies and highlight the benefit of using a standardized dataset. Although GEDM algorithms were the same, variations in validation techniques resulted in different GED accuracies. Zeni et al. compared gait event times detected by CBTA between motion capture data collected at 60 Hz vs. force plate data collected at 600 Hz [12]. Our study performed the same comparison of gait events detected by CBTA vs. force but data were collected at higher sampling rates for both kinematic (128 Hz) and kinetic (3200 Hz) signals. Zeni reported differences of −17.36 ms (right IC), −15.03 ms (left IC), −0.37 ms (right TC), and 11.69 ms (left TC) [12] while our analysis resulted in average differences of −26.78 ms (IC) and −2.49 ms (TC). While results differed in absolute value, the direction matched their previous validation demonstrating that CBTA detected right/left IC and right TC before GS. In another study, IC and TC RMSEs were 26 and 25 ms, respectively, when SK was applied to gyroscope signals and compared to gait event times detected by footswitches [27], whereas, IC and TC RMSEs were 26.53 and 62.44 ms, respectively, when SK GEDM was validated with a motion capture system and force plates in our work. Lastly, two populations (TD and CP) were included in the comparison of gait event times detected by FVA vs. force plate to the literature. Similar to the data collection for CBTA validation, our kinematic and kinetic dataset was collected at higher sampling rates. O’connor et al. demonstrates that IC and TC were detected by FVA 16 ± 15 ms and 9 ± 15 ms later than determined by force plate data, respectively, in TD while IC and TC were detected by FVA 3 ± 9 ms and 6 ± 26 ms earlier in CP [11]. We report that FVA detects IC later in both TD (39.9 ± 22.67 ms) and CP (77.9 ± 164.9 ms) and detects TC earlier in both groups (TD: −3.19 ± 15.53 ms, CP: −20.68 ± 58.48 ms) compared to force plate data. In general, we report gait event timing errors of similar magnitudes and directions as previous investigations. The direct comparison of GED timing of multiple GEDMs in the same dataset illustrates the variation in amount of delay compensation needed and may facilitate sensor selection.

Statistical modeling was employed to systematically assess the influence of covariates such as gait event, subject group, side, and subject-specific differences on the differences observed between the kinematic GEDMs and the GS. Individual models were created for each of the kinematic GEDMs and incorporated adjustments for the additive covariates (group, gait event, side, and subject). The full model performed well for each of the kinematic GEDMs and provided values for comparison of RMSEs for the reduced and final models. Final models containing only the principal predictor (CBTA, SK, or FVA) and random coefficients grew <10 ms RMSE of the full models. The small RMSE difference demonstrates the large contribution that GEDM has on gait event timing differences compared to GS. The RMSE differences of 5 ms or less between the *marginal* model (excludes subject-specific adjustments) and the *conditional* model (includes subject-specific adjustments) at each level of model progression illustrate the robustness of each GEDM to subject differences. The final model shows that only the principal predictor and random coefficients are needed to have the best approximate gait events compared to GS.

Using the RMSE for model comparison indicates little difference between the full model with all predictors and a reduced model with a principal predictor +IC, however, given the relative small effect size for IC, one might question the practical advantage of that model over a simple linear regression using the marginal model coefficients for each of CBTA, SK, and FVA. We recommend the more parsimonious simple linear models with the statistically significant intercepts and slopes appearing as the final marginal model for each measure in Table 4. Results from our models, inclusive of adults and children with and without CP, suggest that timing differences of our data and that of the literature can be accounted for by the RMSEs of the method employed. A conditional RMSE that improves the marginal RMSE less than $10^{1}$ ms suggests that these models can be confidently applied to a wide range of gait characteristics without tuning to subject population characteristics. From a statistical perspective, the tradeoffs between models are minimal. Depending on the desired precision of gait event detection timing, however, the error differences need to be evaluated to determine if they are clinically relevant when applied to gait analysis or rehabilitation applications. Thus, the choice of model and variables included in gait event detection timing compensations may have a small, but meaningful impact upon application.

Any one of the three kinematic GEDMs can be translated into sensor-based systems for gait event detection [28] as they all showed high gait detection reliability of IC and TC. However, not all kinematic GEDMs are equally practical for implementation. Despite the GED accuracy of the CBTA, implementation of this GEDM requires a relatively high number of sensors and sophisticated arithmetic processing. Seven inertial measurement units were used in a CBTA-based sensor system to generate the necessary input signals for GED as well as other spatiotemporal parameters [43]. The number of sensors may be reduced, however, depending the desired output. For example, CBTA-based sensor system may only require three IMUs if only bilateral GED is required. This GEDM may be useful as an alternative for laboratory-based gait analysis, however, it may not be computationally and cost efficient for wearable applications such as orthotics and prosthetics.

Foot velocity (FVA) and shank angular velocity (SK) GEDMs are more easily applied to wearable sensor signals and are commonly used in research laboratories [5,21,44]. Foot velocity can be captured via shoe/foot-attached accelerometers, requiring a minimal sensor setup of one sensor on each side [28], by integrating acceleration over time. A source of GED timing error is the potential drift introduced with signal integration. Sophisticated techniques, such as zero velocity update [45], extended Kalman filters [46] combined with sensor fusion [47], however, may be used to reduce drift. In addition to timing errors introduced from the signal, previous studies have reported that FVA is not applicable to clinical cases [11,48]. Pathologic gait, as demonstrated in CP, does not have a regular heel to toe progression; therefore, difficulty in detecting gait events with non-kinetic based methods is a limitation of this GEDM. If adjustments cannot be made for missing the trough in the foot velocity signal (Figure 1), it can result in decreased detection reliability.

We have identified SK, out of the three GEDMs evaluated in this study, as the kinematic-based method that can best be used for sensor-based gait event detection. The SK GEDM can be applied to signals collected via shank-attached gyroscope sensors (one sensor on each side). Gyroscopes are easy to use, miniature in size and can be used with FES systems, exoskeletons and other clinical/research training systems [5,16,21,49]. This minimal sensor set up facilitates implementation (similar to FVA), is of modest cost, and computationally efficient in wearable applications. Unlike CBTA, minimal processing is required to condition the sensor signals for the SK detection algorithm providing improved processing capacity and enabling implementation of more sophisticated control algorithms in the system [50]. The ample amount of data on reliability and performance of the SK GEDM evaluated in patient populations such as amputees [51], spinal cord injuries [49], and post-stroke survivors [52] demonstrate its robustness to variations in gait. The SK GEDM includes the advantages of the other two GEDMs, such as minimal sensor set (similar to FVA) and comparable detection accuracy and reliability to CBTA, as well as demonstrated GED ability in multiple patient populations.

A limitation of this study is the assumption that GEDM performance in a motion capture system translates to equivalent performance in a sensor-based system. We have demonstrated the advantage of assessing GEDM performance on the same dataset. Previous work reported real-time GED performance using SK against force plates in adults, typically developing children, and children with CP during treadmill walking [5,50]. Gait detection reliability was higher when SK was applied to sensor signals (AD: 99.8% [50], TD: 99.9%, CP: 99.6% [5]) than to motion capture signals (AD: 96.7% TD: 96.3% CP: 95.2%). Comparisons of SK GEDM accuracies between the sensor system (gyroscope signal) and motion capture system are outlined in Table 5. Although gait event detection had a greater RMSE in the sensor system, with the exception of TC in AD, the range of delay was similar (sensor: 49 ms, motion capture: 40 ms) and smaller than the FVA range (166.21 ms).

Other study limitations include small group sizes, although a large number of gait cycles were included in analysis, GEDM performance was isolated to treadmill walking, and we did not account for speed variation. The walking speeds were similar for the AD and TD groups (AD: 0.92 ± 0.18 m/s, TD: 0.99 ± 0.17 m/s), while individuals with CP walked slower (CP: 0.72 ± 0.15 m/s). Exclusion of one subject in the CP group from analysis indicates that kinematic GEDM, and wearable sensors, may be challenging to use in patient populations with more severe gait deviations (discussed above). Further investigation is required to fine-tune our model for these patient populations.

## 5. Conclusions

Three kinematic gait event detection methods (CBTA, SK, FVA) for detecting gait events (IC and TC) during walking were evaluated in three populations (AD, TD, and CP). A standardized dataset was used to compare GEDM performance to the GS and illustrated the differences in detection accuracy and reliability of each GEDM. Covariates were systematically assessed using statistical modeling which showed that GEDM contributed the most to GED accuracy and kinematic-based GEDMs were relatively insensitive to covariates of subject group differences, person-specific variability, and side-to-side gait differences. The SK GEDM was identified an accurate, robust, reliable and easy to implement method in sensor-based systems. Future work will explore leveraging the statistical models to design GED timing compensation algorithms.

## Figures and Tables

**Table 1:** Average initial and terminal contact time difference (mean ± SE) of kinematic versus kinetic gait event detection methods (GEDM) in milliseconds (ms). CBTA: coordinate-based treadmill algorithm; SK: shank angular velocity algorithm; FVA: foot vertical acceleration algorithm.

Gait Event	CBTA		SK		FVA	
	Mean	SE	Mean	SE	Mean	SE
Initial contact	−21.54	0.66	−10.45	0.74	49.50	3.43
Terminal contact	2.47	0.96	−56.20	1.02	−6.88	1.33

**Table 2:** Root mean square error (RMSE) of gait event detected timing using kinematic versus kinetic method in milliseconds (ms). CBTA: coordinate-based treadmill algorithm; SK: shank angular velocity algorithm; FVA: foot vertical acceleration algorithm; AD: adult; TD: typically developing children; CP: children with cerebral palsy.

Gait Event	Group	CBTA	SK	FVA
Initial contact	AD	33.10	26.53	42.06
	TD	27.53	22.55	44.13
	CP	20.43	17.06	182.03
Terminal contact	AD	14.98	62.44	14.76
	TD	27.79	58.69	15.82
	CP	35.09	66.73	61.91

**Table 3:** Percent gait detection reliability (GDR) of gait event detection methods (GEDM).

		Kinetic	Kinematic		
Gait Event	Group	GS	CBTA	SK	FVA
Initial contact	AD	100.0	95.3	95.9	96.6
	TD	99.5	97.3	95.5	95.0
	CP	99.2	94.8	94.8	87.1
Terminal contact	AD	99.7	95.6	97.5	96.2
	TD	100.0	96.8	97.2	95.9
	CP	100.0	95.1	95.5	95.1

**Table 4:** Model development progression.

Model	Marginal Coefficient	Standard Error	*p*-Value	Marginal RMSE (ms)	Conditional RMSE (ms)
Full (CBTA)				20.00	18.93
CBTA	0.99999	0.00006	<0.0001		
Intercept	7.78694	4.82735	0.1262		
Reduced (CBTA+IC)				22.49	19.16
CBTA	0.99998	0.00006	<0.0001		
IC	24.0017	0.99473	<0.0001		
Intercept	−2.98337	3.40686	0.3985		
Final (CBTA)				25.50	22.61
CBTA	1.00001	0.00007	<0.0001		
Intercept	8.63979	3.41579	0.0298		
Full(SK)				24.14	21.82
SK	0.99994	0.00005	<0.0001		
Intercept	55.0062	5.34258	<0.0001		
Reduced (SK+IC)				24.52	21.87
SK	0.99994	0.00005	<0.0001		
IC	−45.7127	1.12596	<0.0001		
Intercept	56.2336	2.89395	<0.0001		
Final (SK)				33.53	32.03
SK	0.99996	0.00008	<0.0001		
Intercept	33.3328	2.91872	<0.0001		
Full (FVA)				70.02	65.07
FVA	0.99989	0.00024	<0.0001		
Intercept	13.4269	13.7265	0.3442		
Reduced (FVA+IC)				70.10	65.10
FVA	0.99989	0.00024	<0.0001		
IC	−57.4868	3.41134	<0.0001		
Intercept	10.7528	7.46160	0.1689		
Final (FVA)				75.56	71.19
FVA	0.99994	0.00026	<0.0001		
Intercept	−18.3814	7.15659	0.0222		

**Table 5:** Root mean square error (RMSE) of gait event detection (ms) of shank angular velocity versus gold standard for sensor and motion capture systems. The sensor system used a gyroscope signal [5,50]. AD: adult; TD: typically developing children; CP: children with cerebral palsy.

Gait Event	Group	Sensor	Motion Capture
Initial contact	AD	32	27
	TD	52	23
	CP	63	17
Terminal contact	AD	33	62
	TD	70	59
	CP	81	67

## Appendix A

Model diagnostics identified a pattern of residuals inconsistent with model assumptions, largely attributable to a subject in the CP group. Plots representing residuals by order, color-coded by subject order suggest that residuals from this single subject with CP were unusual (blue dots) with respect to other subjects and both created an upward bias in the RSME and caused the overall pattern of residuals to depart from normality (Figure A1).

This individual with CP had inconsistent scissoring across midline, exhibited dragging of the toes, and had a lower functioning GMFCS level III. This subject used a rollator walker during over-ground walking and walked with a shorter step width (0.086 m), stride length (0.648 m), increased hip flexion throughout gait, increased knee flexion from midswing to midstance and increased ankle dorsiflexion. These gait deviations contributed to misdetections in the standard kinetic force plate detection method as well as for the three kinematic methods. This individual was excluded from the analysis.
